# Genotypic Characterization of Azotobacteria Isolated from Argentinean Soils and Plant-Growth-Promoting Traits of Selected Strains with Prospects for Biofertilizer Production

**DOI:** 10.1155/2013/519603

**Published:** 2013-11-03

**Authors:** Esteban Julián Rubio, Marcela Susana Montecchia, Micaela Tosi, Fabricio Darío Cassán, Alejandro Perticari, Olga Susana Correa

**Affiliations:** ^1^Instituto de Microbiología y Zoología Agrícola (IMYZA), Instituto Nacional de Tecnología Agropecuaria (INTA), Dr. Nicolás Repetto y De los Reseros s/n, 1686 Hurlingham, Buenos Aires, Argentina; ^2^Cátedra de Microbiología Agrícola/INBA (CONICET/UBA), Facultad de Agronomía, Universidad de Buenos Aires, Av San Martín 4453, C1417DSE Ciudad Autónoma de Buenos Aires, Argentina; ^3^Laboratorio de Fisiología Vegetal, Departamento de Ciencias Naturales, Facultad de Ciencias Exactas, Físico-Químicas y Naturales, Universidad Nacional de Río Cuarto, Ruta 8, Km 601, 5800 Río Cuarto, Córdoba, Argentina

## Abstract

The genetic diversity among 31 putative *Azotobacter* isolates obtained from agricultural and non-agricultural soils was assessed using rep-PCR genomic fingerprinting and identified to species level by ARDRA and partial 16S rRNA gene sequence analysis. High diversity was found among the isolates, identified as *A. chroococcum*, *A. salinestris*, and *A. armeniacus*. Selected isolates were characterized on the basis of phytohormone biosynthesis, nitrogenase activity, siderophore production, and phosphate solubilization. Indole-3 acetic-acid (IAA), gibberellin (GA_3_) and zeatin (Z) biosynthesis, nitrogenase activity, and siderophore production were found in all evaluated strains, with variation among them, but no phosphate solubilization was detected. Phytohormones excreted to the culture medium ranged in the following concentrations: 2.2–18.2 **μ**g IAA mL^−1^, 0.3–0.7 **μ**g GA_3_ mL^−1^, and 0.5–1.2 **μ**g Z mL^−1^. Seed inoculations with further selected *Azotobacter* strains and treatments with their cell-free cultures increased the number of seminal roots and root hairs in wheat seedlings. This latter effect was mimicked by treatments with IAA-pure solutions, but it was not related to bacterial root colonization. Our survey constitutes a first approach to the knowledge of *Azotobacter* species inhabiting Argentinean soils in three contrasting geographical regions. Moreover, this phenotypic characterization constitutes an important contribution to the selection of *Azotobacter* strains for biofertilizer formulations.

## 1. Introduction

The genus *Azotobacter*, which belongs to the family Pseudomonadaceae from the subclass *γ*-Proteobacteria, comprises seven species: *Azotobacter vinelandii*, *A. chroococcum*, *A. salinestris*, *A. nigricans*, *A. beijerinckii*, *A. paspali*, and *A. armeniacus* [1]. Azotobacteria are aerobic, heterotrophic, and free-living N_2_-fixing bacteria, which can be isolated from soil, water, and sediments [2]. Several studies have demonstrated that seed inoculation with *Azotobacter* improves maize [3], wheat [4, 5], and rice [6] yields. However, although there is a considerable amount of experimental evidence of these positive effects on plant growth, mechanisms involved are not fully understood. The ability to fix N_2_ was the main feature leading to the use of *Azotobacter* as a biofertilizer in the past. Nowadays, however, it is well established that non-symbiotic fixation can improve plant growth only indirectly, by increasing soil nitrogen after mineralization of N2-fixers' biomass. More likely, additional abilities of azotobacteria, such as phosphate solubilization and phytohormone and siderophore synthesis, might contribute more directly to increase plant growth and crop yield [4, 7, 8].

Like many plant-growth promoting bacteria, azotobacteria have the capacity to excrete auxins to the culture medium. Auxins and indole-3 acetic-acid (IAA) as the most common member of auxin family were the first plant hormones to be discovered and are implicated in virtually every aspect of plant growth and development. It has been reported that inoculation with auxin-releasing *Azotobacter* strains increases growth, yield, and nitrogen uptake in wheat and maize and that the combined application of *Azotobacter* and tryptophan, which is often implicated in IAA synthesis, enhances plant growth in a greater extent [5, 9, 10]. These results suggest that auxin production might be a key mechanism of *Azotobacter* in promoting plant growth and yield, as it has been reported in other bacteria.

The importance of studying plant-growth promoting bacteria (PGPR) lies on their potential to be used as biofertilizers. The use of biofertilizers containing living microorganisms is a welcoming management alternative in sustainable systems, like organic and low-input agriculture, as well as a tool to reduce the use of chemicals in intensive agriculture [11]. When formulating a biofertilizer, it is highly recommended to consider the use of native bacteria, because they are better adapted to ecological conditions and, therefore, are more competitive than nonnative strains [5]. Hence, the isolation and characterization of native bacterial strains should be one of the first steps when developing commercial biofertilizers. In Argentina, the diversity of *Azotobacter* in soils has not yet been studied and any *Azotobacter*-based biofertilizers have been developed.

For the above mentioned facts, the aims of our study were to isolate and characterize *Azotobacter *strains from agricultural and non-agricultural soils, covering a wide range of geographic regions and soil types, and to study some bacterial traits involved in plant growth stimulation. To test this, we first assessed genetic diversity among isolates by repetitive sequence-based PCR genomic fingerprinting (rep-PCR) and identified them by amplified ribosomal DNA restriction analysis (ARDRA) and partial 16S rRNA gene sequence analysis. Then, some of these isolated strains were tested for hormone biosynthesis (indole-3-acetic acid (IAA), gibberellic acid (GA_3_), and zeatin (Z)), siderophore production, nitrogen fixation capacity, and phosphate solubilization. Finally, we tested early-growth stimulation of wheat roots by inoculation with some of the isolated *Azotobacter* strains.

## 2. Materials and Methods

### 2.1. Soil Sampling, Bacterial Isolation, and *Azotobacter* Reference Strains

In total, 74 bulk soil samples (0–20 cm) were collected from agricultural (53 samples) and non-agricultural sites (21 samples) during spring 2006. Samples belonged to 38 different locations of Northwest, Pampas, and Patagonia regions of Argentina (see Supplementary Material available online at http://dx.doi.org/10.1155/2013/519603). Soil aggregates (~2 mm) were spread onto the surface of Petri dishes containing N-free Burk's agar medium with mannitol as C-source [1]. After five days at 28°C, slimy and glistening *Azotobacter*-like colonies growing around soil particles were selected and further purified in N-free LG with bromothymol blue agar medium [1]. Motility, pigment production, and encystment were determined as previously described [1]. Isolates were preserved at −80°C in Burk's medium [1] with 30% (v/v) glycerol.

*Azotobacter vinelandii* reference strains (NRRL B-14627, NRRL B-14641, and NRRL B-14644) were obtained from the ARS Culture Collection (NRRL), USA, and *A. chroococcum* reference strain BNM 272, isolated from Argentinian soils, was provided by the Banco Nacional de Microorganismos, Argentina.

Electrical conductivity (EC), organic matter (OM), pH, and extractable phosphorus of the soils samples were determined at the Instituto de Suelos (INTA, Buenos Aires, Argentina) using standard procedures [12].

### 2.2. Rep-PCR Genomic Fingerprinting

Repetitive sequence-based PCR genomic fingerprints of isolates were obtained with BOX-A1R primers [13] as previously described [14], by using 1-*μ*L portions of whole-cell suspensions of each isolate as templates. Fingerprints were analyzed using GelCompar II v. 6.5 (Applied Maths NV). Dendrogram was elaborated based on Pearson's correlation coefficient and the UPGMA algorithm.

### 2.3. Amplified Ribosomal DNA Restriction Analysis (ARDRA)

Representative strains of each rep-PCR cluster were analyzed by ARDRA, as previously described [2], using the primers fD1 and rD1 and the restriction enzymes *Rsa*I or *Hha*I. ARDRA profiles were analyzed with GelCompar II and compared using the Dice similarity coefficient to construct the similarity matrix. The dendrogram was obtained by UPGMA.

*In silico* ARDRA was carried out with *Hha*I using the restriction mapper software (http://www.restrictionmapper.org/) and 16S rRNA gene sequences AB175656 (*A. salinestris* ATCC 49674^T^) and FJ032010 (*A. salinestris* I-A), both obtained from GenBank.

### 2.4. 16S rRNA Gene Sequencing

The partial 16S rRNA gene sequence was amplified using primers Y1 and Y2 [15]. Then, amplicons (~290 bp) were purified using the QIAquick PCR purification kit (Qiagen, GmbH) and sequenced by Unidad de Genómica (Instituto de Biotecnología, INTA, Buenos Aires, Argentina) in both directions using the same primers. The obtained sequences were compared with those from GenBank using BLASTN 2.2.16 [16].

### 2.5. Nucleotide Sequence Accession Numbers

The obtained 16S rRNA gene sequences were deposited at the GenBank/EMBL/DDBJ database under the following accession numbers: HQ541448, HQ591467, HQ623180, HQ623181, HQ623182, HQ623178, and HQ623179.

### 2.6. Determination of Potential Plant Growth-Promoting Traits

Eighteen selected strains were assessed for siderophore production according to the O-CAS method [17]. Phosphate-solubilizing activity was tested on Pikovskaya medium [18], NBRIP medium [19] and modified Burk's agar medium [1], adding 0.5% of Ca_3_(PO_4_)_2_ to each medium as insoluble P source. In both assays, *Pseudomonas fluorescens* BNM233 (Banco Nacional de Microorganismos, Buenos Aires, Argentina) was used as a positive control.

Auxin production was determined using a colorimetric assay [20], with measurements after 1, 2, 3, and 5 days of growth in modified LG (LGSP) liquid medium containing 1% sucrose and 0.5% soymeal peptone. At each time interval, the number of cells (cfu mL^−1^) was determined by plate counting on LG agar.

Nitrogenase activity was estimated by the acetylene reduction assay. Bacterial cultures were grown in N-free Burk's agar medium at 28°C for 24 h and ethylene production was measured by gas chromatography [21], using a Hewlett Packard Series II 5890 equipped with a flame ionization detector (FID) and a stainless-steel Porapak N column (3.2 mm × 2 m; 80/100 mesh). The injector, oven, and detector temperatures were 110°C, 90°C, and 250°C, respectively. N_2_ was used as carrier gas (4.5 cm s^−1^ linear gas velocity). Total protein concentration of bacterial cells was determined by the Lowry method with the DC Protein Assay kit (Bio-Rad, USA). Nitrogenase activity was expressed as mmol ethylene produced per mg of protein in 24 h.

Indole-3-acetic acid (IAA), gibberellic acid (GA_3_), and zeatin (Z) production were determined for six selected *Azotobacter* spp. strains grown in LGSP liquid medium at 28°C for 8 days. Z was identified and quantified by HPLC-UV, whereas IAA and GA_3_ were identified by gas chromatography-mass spectrometry with selective ion monitoring (GC-MS-SIM), as previously described [21].

### 2.7. Effects of *Azotobacter* Inoculation and IAA Pure Solutions on the Number of Seminal Roots and Root Hairs of Wheat Seedlings

For plant tests, seeds of wheat (*Triticum aestivum* cv. Baguette Premium 13, Nidera, Buenos Aires, Argentina) were surface-disinfected (1% NaClO for 3 minutes) and germinated in plastic containers (15 × 25 × 4 cm) on filter paper soaked with sterile distilled water. To maintain humidity, containers were wrapped in transparent plastic bags and placed in a growth chamber at 25°C with a 16 h light/8 h dark regime for 24 h. For inoculation, bacterial strains were grown in LGSP liquid medium at 28°C for 8 days (~10^8^ cfu mL^−1^). Fifteen pregerminated seeds were inoculated with 100 *μ*L of bacterial culture (~10^7^ cells) per seed and grown for 8 days as described above. Eight treatments were applied: (a) control (100 *μ*L of sterile distilled water); (b) and (c) two phytohormone treatments based on 100 *μ*L of low (2 *μ*g mL^−1^) and high (20 *μ*g mL^−1^) concentrations of pure-IAA solutions (Sigma-Aldrich), sterilized by filtration (0.2 *μ*m filter); (d) *A. salinestris *AT18; (e) *A. salinestris* AT37; (f) *A. salinestris* AT19; (g) *A. chroococcum *AT25; and (h) *A. chroococcum* AT31. Treatments were run in triplicate (three containers each). For bacterial root colonization, roots of two plants per container (a total of six plants per treatment) were ground in 2 mL of sterile distilled water with mortar and pestle. Serial dilutions were inoculated in triplicate on LG agar plates and incubated at 28°C for 72 h. At the end of the experiment, root colonization (cfu per root of *Azotobacter*-like colonies) and number of seminal roots were determined. Two independent experiments were run.

The effects on root tip morphology of cell-free culture of two selected *A. salinestris *strains (AT18 and AT19) with different levels of phytohormone production (Figure 3) and root colonization (Table 3) but similar nitrogenase activity (Figure 3) were assessed and compared to the application of two IAA-pure solutions, 2 and 20 *μ*g mL^−1^. Fifteen pre-germinated wheat seeds per treatment were placed in three Petri dishes (five seeds per dish) containing 0.7% water agar. Seedling treatments were as follows: (a) control (100 *μ*L of sterile distilled water), (b) 100 *μ*L of 2 *μ*g mL^−1^ IAA-pure solution, (c) 100 *μ*L of 20 *μ*g mL^−1^ IAA-pure solution, (d) 100 *μ*L of *A. salinestris* AT18 cell-free culture, and (e) 100 *μ*L of *A. salinestris* AT19 cell-free culture. After 4 days at 25°C under dark conditions, seedling roots were stained with crystal violet solution (0.075% in 70% ethanol) and observed in a binocular microscope at 25x.

### 2.8. Experimental Design and Data Analysis

Each inoculation experiments were performed in a complete randomized design. Data were analyzed by ANOVA and DGC multiple comparisons *post hoc* analysis [22] (*α* = 0.05), using INFOSTAT software [23].

## 3. Results

### 3.1. *Azotobacter* Isolates Obtained from Argentinean Soils and Chemical Parameters of Soils

We isolated *Azotobacter*-like bacteria from 23 soil samples (11 agricultural and 12 non-agricultural soils) from a total of 74 screened samples (Table 1 and Supplementary Material). Isolates were obtained from soils with a wide range of values for organic matter content (0.19–5.72%), pH (5.8–8.7), electrical conductivity (0.2–2.2 mS cm^−1^), and extractable phosphorus (1.9–127.8 ppm) (Table 1). 

We obtained 31 bacterial isolates that were preliminary characterized on the basis of pigment production and cell morphology. All of them produced nondiffusible brown pigments in agar medium, showed motile cells, formed cysts in butanol-containing medium, and showed no fluorescent pigments under UV light (data not shown).

### 3.2. Genomic Fingerprinting by rep-PCR

**Figure 1:**
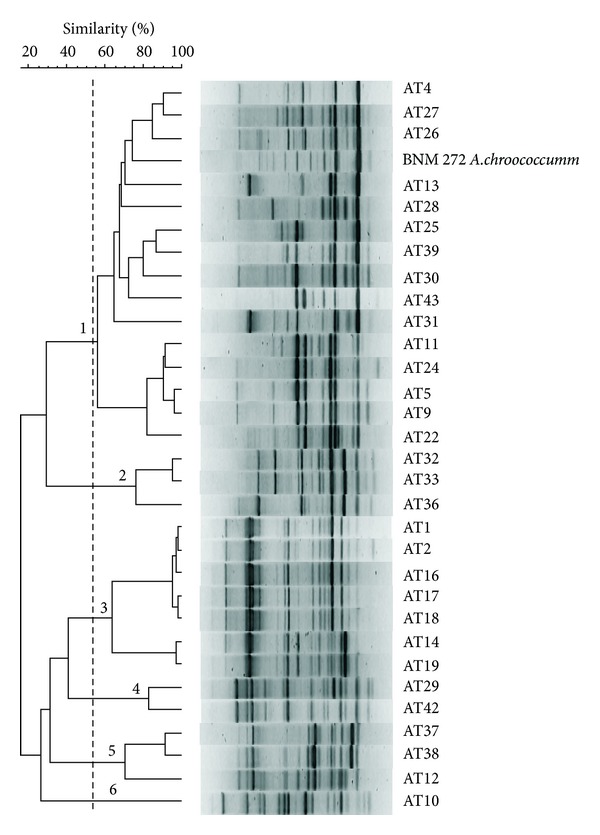
Genetic diversity of azotobacteria isolated from agricultural and non-agricultural soils from different regions of Argentina revealed by rep-PCR genomic fingerprinting analysis. The dendrogram was constructed by using the Pearson correlation coefficient (*r*) and the UPGMA method using GelCompar II version 6.5 software. The groups indicated by 1 to 6 numbers were defined at the 55% similarity level (vertical dashed line). The cophenetic correlation value for this dendrogram was 0.92.

The intraspecific diversity among 31 isolates was assessed by means of rep-PCR. Most isolates showed distinctive banding profiles, reflecting the genetic diversity among them. The cluster analysis of fingerprints revealed six major groups among all isolates at 55% similarity level (Figure 1). Isolates showing highly similar fingerprints (similarity > 90%) were considered clonemates. As a result, 23 distinct strains were obtained. No clear relationship could be established between rep-PCR clustering and the geographical origin of isolates. For example, group 1 included strains which were isolated from four provinces (Buenos Aires, Chubut, Entre Ríos, and Jujuy) of the three regions (Pampas, Northwest, and Patagonia). However, some tendencies between clustering and the origin of soil samples were observed. Group 2 clustered all isolates from Córdoba province (Pampas region), group 3 included strains isolated from Salta and Santiago del Estero provinces (Northwest region), and group 4 included two strains obtained from Chubut province (Patagonia region) (Figure 1 and Table 1). We chose representative strains of each group to classify them using ARDRA.

### 3.3. ARDRA and 16S rRNA Gene Sequence Analysis

**Figure 2:**
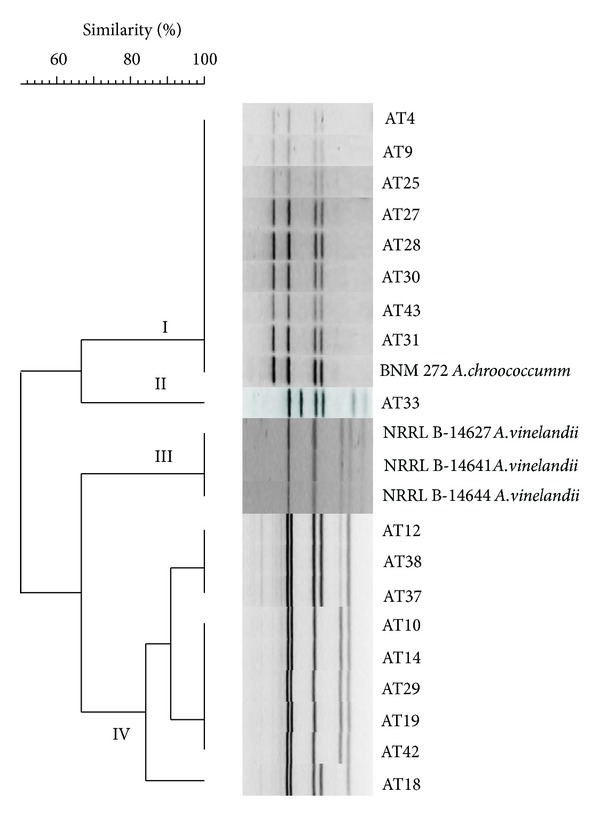
Amplified ribosomal DNA restriction analysis (ARDRA) of* Azotobacter* representative strains of each rep-PCR group and reference strains. The dendrogram based on analysis of restriction patterns of 16S rDNA obtained with *Hha*I was built using the GelCompar II program and the Dice (*S*_*D*_) pairwise coefficient of similarity and the UPGMA algorithm. Clusters were defined at the *S*_*D*_ > 80% similarity level. The cophenetic correlation value for this dendrogram was 0.95.

ARDRA with *Rsa*I and *Hha*I restriction enzymes was used to identify *Azotobacter *strains to genus and species level, as previously recommended for the molecular identification of these microorganisms [24]. The 18 chosen strains represented, altogether, the six rep-PCR clusters. All strains yielded single amplification products of the expected size (about 1,500 bp) for the 16S rRNA genes and showed identical restriction *Rsa*I profiles (data not shown), characteristic of the genus *Azotobacter* [2, 24]. When ARDRA was performed using* Hha*I, six different profiles were obtained. Cluster analysis of *Hha*I restriction profiles revealed four distinct clusters at 80% similarity level (Figure 2). Since all strains grouped in cluster I showed profiles distinctive of *A. chroococcum*, as reported by Aquilanti et al. 2004 [2], and identical to those of *A. chroococcum* reference strain BNM 272, they were assigned to this species. Cluster II included only strain AT33, which showed a characteristic banding profile of the species *A. armeniacus* [2], whereas cluster III contained only the three *A. vinelandii* strains used as reference. The ARDRA profiles of strains in cluster IV, obtained experimentally, were similar to those of *A. salinestris* reference strains ATCC 49674^T^ and I-A done *in silico*. According to these results, the strains of heterogeneous cluster IV (Figure 2) were assigned to *A. salinestris*.

To confirm species identification of isolates, partial sequencing of the 16S rRNA gene was performed for seven strains representing ARDRA clusters. Based on the similarity observed among these sequences, strains AT25 and AT31 in cluster I (Figure 2) were related to *A. chroococcum* LMG 8756^T^ (99% identity), strain AT33 in cluster II was related to *A. armeniacus* DSM 2284^T^ (99% identity), and the four strains in cluster IV (AT18, AT19, AT37, and AT42) were related to *A. salinestris* ATCC 49674^T^ (99-100% identity).

Summarizing, according to the results obtained by rep-PCR, ARDRA, and partial sequencing of the 16S ribosomal gene, the 15 isolates of group 1 of rep-PCR (Figure 1) were classified as *A. chroococcum*, the three isolates of group 2 as *A. armeniacus*, and the 13 isolates included in groups 3 to 6 as *A. salinestris*.

### 3.4. Siderophore and Phytohormone Production, Phosphate Solubilization, and Nitrogenase Activity

All the 18 strains tested exhibited a color change from blue to orange in CAS medium, which is indicative of siderophore production. Phosphate-solubilizing activity was not evident in any of the *Azotobacter* strains assayed, independently of the medium used (data not shown). All preselected strains were assayed for auxin production in LGSP medium using the Salkowski reagent method. After one day of growth (~10^8^ cfu mL^−1^), all bacterial strains produced low levels of auxin (0.96 *μ*g mL^−1^ to 2.64 *μ*g mL^−1^) (Table 2). An important increase was observed after two and three days of growth, without any changes in cfu mL^−1^ (data not shown). Finally, bacterial strains differed in the levels of auxin excreted to the culture medium at the end of the assay, covering a range of values from 2.2 to 19.5 *μ*g mL^−1^ (Table 2). *A. salinestris* AT12, AT14, AT19, and AT29 and *A. chroococcum* AT25, AT30, AT31, and AT39 reached up to a ~10-fold increase from the first to the fifth day (Table 2). No changes in the number (cfu mL^−1^) of bacteria were observed at the end of the assay (data not shown).

Using these results, the 18 *Azotobacter* strains were arbitrarily classified as low- (2–6 *μ*g mL^−1^), medium- (7–14 *μ*g mL^−1^), and high- (>14 *μ*g mL^−1^) auxin producers (Table 2). Then, we selected three low-auxin-producing strains (AT18, AT37, and AT42) and three high-auxin-producing strains (AT19, AT25, and AT31) and assessed them in nitrogen fixing capacity and biosynthesis of three phytohormones (IAA, GA_3_, and Z).

**Figure 3:**
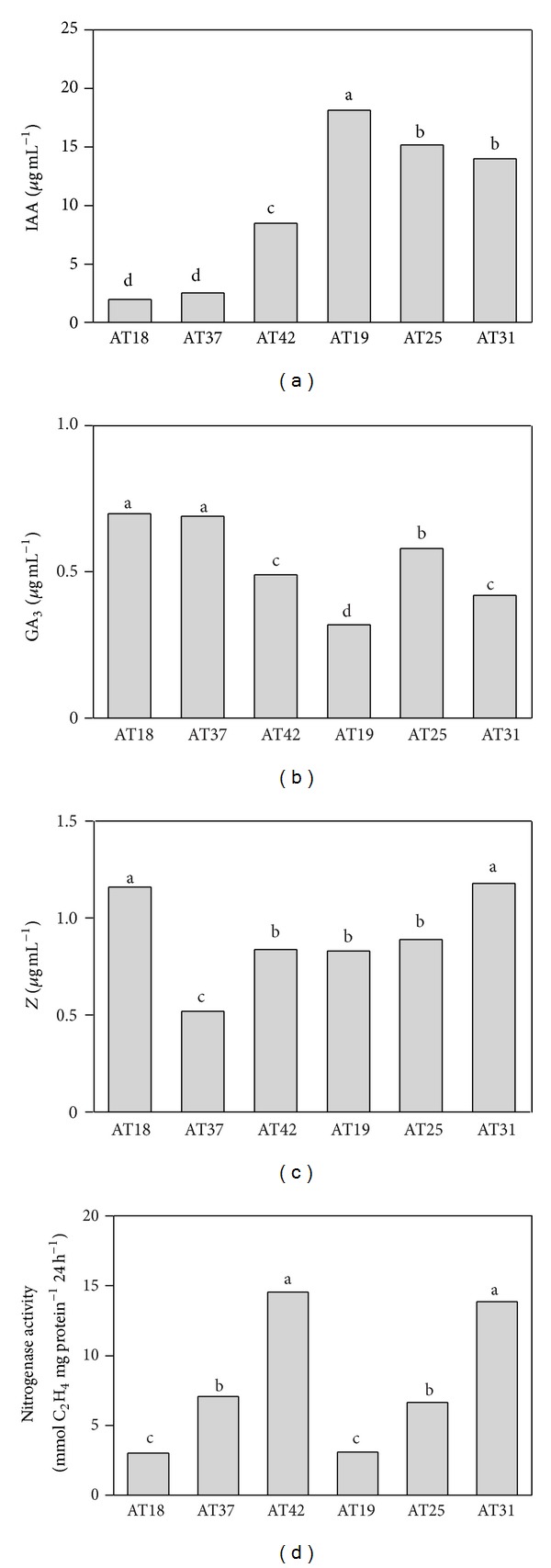
Phytohormone production and nitrogenase activity by the selected *Azotobacter* strains. (a) Indole-3-acetic acid (IAA) production; (b) gibberellic acid (GA_3_) production; (c) zeatin (Z) production, and (d) nitrogenase activity. IAA and GA_3_ were identified and quantified by gas chromatography-mass spectrometry, Z was identified and quantified by HPLC-UV, and nitrogenase activity (acetylene-ethylene reduction) was determined by gas chromatography. Bars are means of three replicates. The same letters indicate no significant differences between means as determined by the DGC test (*P* = 0.05).

Concerning the nitrogenase activity, the highest activity levels (~14 mmol C_2_H_4 _mg protein^−1^ 24 h^−1^) were exhibited by *A. salinestris *AT42 and *A. chroccoccum* AT31 strains. *A. salinestris* AT37 and *A. chroccoccum* AT25 strains presented intermediate levels (6.5 mmol C_2_H_4 _mg protein^−1^ 24 h^−1^), and the lowest values (3 mmol C_2_H_4 _mg protein^−1^ 24 h^−1^) were found in *A. salinestris* AT18 and AT19 strains (Figure 3(d)).

*A. salinestris* AT19 produced the highest level of IAA (18.2 *μ*g mL^−1^), the lowest level of GA_3_ (0.3 *μ*g mL^−1^), and an intermediate value of Z (0.8 *μ*g mL^−1^). By contrast, *A. salinestris* AT18 and AT37 showed the lowest levels of IAA production (2.2–2.6 *μ*g mL^−1^) and the highest levels of GA_3_ production (0.7 *μ*g mL^−1^). These two strains, however, differed in their Z synthesis: while AT18 was one of the largest Z producers (1.2 *μ*g mL^−1^), AT37 exhibited the lowest production (0.5 *μ*g mL^−1^). Similar tendencies were observed when strains AT42 and AT31 were compared. Striking results were obtained with *A. chroccoccum *strain AT25, whose production of the three phytohormones was always in intermediate levels (Figures 3(a), 3(b), and 3(c)). A strong agreement was observed between auxin production measured by the Salkowski reagent method and IAA production determined by GC-MS-SIM, excepting AT42 strain (Table 2 and Figure 3(a)).

### 3.5. Effects of *Azotobacter* Inoculation and IAA Pure Solutions on Root Morphology of Wheat Seedlings

Five strains were used for inoculation assays, where all of them induced a significant increase (on average 17%) in the number of seminal roots of wheat seedlings (Table 3). The greatest increase in the number of seminal roots (20%) was obtained when treated with the high IAA-pure solution and inoculating with the three high-IAA-producing strains (*A. chroococcum* AT25 and AT31 and *A. salinestris* AT19). The results of bacterial inoculation did not seem to be related to the colonization of roots by *Azotobacter*. For instance, *A. salinestris* AT37 and *A. chroococcum* AT31 showed similar values of root colonization (on average 7.5 × 10^5^ cfu root^−1^), but the latter was the one showing the largest positive effect on the number of seminal roots. Maybe, a more direct relationship could be established between the stimulation of this feature and the relative amount of phytohormones excreted by the inoculated *Azotobacter* strains (Figures 3(a) and 3(c)).

**Figure 4:**
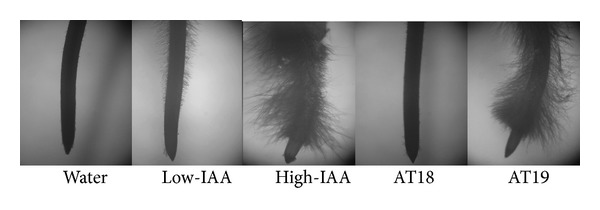
Effect of IAA pure solutions and cell-free cultures of *A. salinestris* treatments on root morphology of 4-day-old wheat seedlings. Root tips of wheat seedlings treated with solutions of 2 *μ*g mL^−1^ and 20 *μ*g mL^−1^ of IAA (low-IAA and high-IAA, resp.) and cell-free cultures of low- (AT18) and high- (AT19) auxin-producing *Azotobacter* strains.

The effect of cell-free culture and IAA-pure solution treatments on the number of root hairs was evaluated on 4-day-old wheat seedlings. Treatments with cell-free culture resulted in a stimulation of root hair number (Figure 4) when compared with control. A higher effect was observed with cell-free culture of AT19 strain than that of AT18 strain. This effect could be mimicked replacing cell-free culture of AT19 strain by the high-IAA (20 *μ*g mL^−1^) pure solution (Figure 4). In contrast, both cell-free cultures of AT18 strain and low-IAA pure solution treatments had a lesser effect on root hair production, compared with the AT19 cell-free culture or the high-IAA solution (Figure 4).

## 4. Discussion

The genotypic characterization of *Azotobacter* native isolates allowed us to identify three *Azotobacter *species and several strains that showed a remarkable diversity. Among the 23 strains isolated from 16 locations in Argentina, including both agricultural and non-agricultural soils, *A. chroococcum* and *A. salinestris* were the species showing the highest frequency (48% and 42%, resp.). This result is in agreement with other studies that reported *A. chroococcum* as the most common species isolated from soils [1, 2, 23]. However, considering that less than a half soil samples contained azotobacteria (23 samples from a total of 74 analyzed soils samples), *Azotobacter* species do not seem to be frequently found in Argentinean soils. Also, the isolation of *Azotobacter *was interestingly more recurrent in non-agricultural than in agricultural soil samples (57% versus 20%, resp.). Even though there are no similar previous reports in the literature, these results may indicate a decrease of azotobacteria in anthropogenically disturbed soils. Hence, the application of biofertilizers with *Azotobacter* might make up, at least partially, the loss of this beneficial bacterial genus in agricultural systems.

The identification of *A. salinestris* and *A. armeniacus* in Argentinean soil samples was a surprising result because, until now, few reports have mentioned the isolation of these species. The presence of *A. salinestris* was reported in soils of western Canada [25], while *A. armeniacus* was reported in soils of Armenia [26]. Although the isolation frequency of both species from soil seems to be low, our results suggest that they might have a more worldwide distribution than thought. Another surprising result was that no *A. vinelandii* strain was isolated in our study, although this species has been reported as a common soil inhabitant [26, 27]. Discrepancies found between our study and earlier reports may be attributed, at least in part, to the identification methodology used. Some misclassifications might have occurred in the past [28] due to the scarcity of genotypic characterizations of *Azotobacter *isolates. In addition, the sources from where the isolates were withdrawn could also explain these differences: in many previous studies, *Azotobacter* strains were isolated from rhizospheric soil, while in this study, the isolates were obtained from bulk soil, a fraction not directly influenced by root activity. Our results reveal the wide tolerance of *Azotobacter* genus to different climate conditions, types of soil, and soil characteristics such as organic matter content, pH values, and phosphorous concentrations.

IAA and GA_3_ production in our collection of *Azotobacter* strains was higher than that reported for a phyllospheric *A. chroococcum* strain REN_2_ [9]. Conversely, other *Azotobacter* strains, isolated from rhizospheric soil in India, reached the same IAA production levels than our high-IAA-producing strains [29]. Although all tested strains excreted phytohormones in chemical complex growing medium, the levels of IAA, GA_3_, and Z production differed among them. Interestingly, IAA production showed high levels in almost all *A. chroococcum* strains but variable levels in *A. salinestris *strains, agreeing with its higher intraspecific diversity revealed by rep-PCR. Even though the production of phytohormones by *A. beijerinckii*, *A. chroococcum*, *A. paspali,* and *A. vinelandii* has been reported by researchers since 1937 [30], as far as we are concerned, this is the first report of *in vitro* phytohormone production by *A. salinestris* strains.

Our results suggest that these isolated *Azotobacter *strains have the potential capacity to promote plant growth directly, through physiological mechanisms such as phytohormone production, in addition to biological nitrogen fixation and siderophore production. The observed changes in root morphology after inoculation with *Azotobacter* or cell-free culture treatment seem to be directly related to the capacity of each strain to synthesize IAA. In previous studies, it was shown that root hairs and seminal roots can be affected by IAA concentration [9, 31]. Nonetheless, it is well known that other phytohormones are involved in regulating plant growth and development. GA_3_ and Z, for instance, have also been previously associated with the stimulation of many aspects of plant growth [32] but, despite this, it is known that plant hormones rarely function alone, and, even in cases in which responses appear to be directly linked to the application of a single hormone, these responses can also be a consequence of other endogenous hormones that are present in plant tissues [32].

## 5. Conclusions

The genotyping of azotobacterial isolates by the combined analysis of ARDRA and rep-PCR and the screening of isolates based on their *in vitro* traits for potential plant growth promoting activity were useful tools for their taxonomic classification and phenotypic characterization. This survey, embracing different regions of Argentina, allowed us to have a first approach to the presence of this bacterial genus in soils. Evaluation of plant growth-promoting traits in bacterial strains is a very important task as criteria for strain selection for biofertilizer formulations. As biofertilizers are a complex resulting from bacteria and their metabolites excreted to the growing medium, it becomes relevant to evaluate every constituent of a biofertilizer before considering it as a potential candidate for field application. Thus, our results constitute an important technological contribution to *Azotobacter* strain selection for biofertilizer formulations that would help to implement a more sustainable agriculture through decreasing the use of agrochemicals.

## Supplementary Material

Supplementary Table: Complete list of all soil samples analyzed. Geographical origin and coordinates, land use, number of Azotobacter-like isolates obtained per soil sample, and soil characteristics.

## Figures and Tables

**Table 1:** Geographical origin and land use of soil samples from which *Azotobacter* isolates were obtained. Summary of fingerprinting and identification results of isolates and soil chemical characteristics.

Geographical origin*	Sampling site	Isolate	rep-PCR group	ARDRA cluster	Partial *16S rDNA* sequence (accession number)	Species assignments	Soil chemical parameters**			
							OM (%)	pH	EC (mS cm^−1^)	P (ppm)
Buenos Aires (Azul)	Maize stubble	AT25	1	I	HQ623180	*A. chroococcum *	3.38	7.30	0.48	7.40
Buenos Aires (Balcarce)	Agricultural bare	AT22	1	nd		*A. chroococcum *	5.72	5.80	1.21	51.00
Buenos Aires (Mar Chiquita)	Lagoon bank 1	AT30	1	I		*A. chroococcum *	1.86	8.20	0.43	1.90
Buenos Aires (Mar Chiquita)	Lagoon bank 2	AT31	1	I	HQ623181	*A. chroococcum *	1.05	8.00	1.45	7.70
Buenos Aires (Santa Clara del Mar)	Urban land	AT4	1	I		*A. chroococcum *	0.98	8.45	0.48	8.50
		AT5	1	nd		*A. chroococcum *				
Buenos Aires (Santa Clara del Mar)	Side of road	AT9	1	I		*A. chroococcum *	5.72	7.83	0.80	8.50
Chubut (Esquel)	Natural pasture	AT24	1	nd		*A. chroococcum *	2.74	6.40	0.49	40.40
Chubut (Gaiman)	Natural pasture	AT28	1	I		*A. chroococcum *	3.15	8.30	0.66	45.80
Chubut (Trevelín)	River bank	AT43	1	I		*A. chroococcum *	1.02	6.60	1.58	8.10
Jujuy (Tilcara)	Side of route^a^	AT11	1	nd		*A. chroococcum *	0.19	8.77	0.28	4.80
		AT13	1	nd		*A. chroococcum *				
Jujuy (Tilcara)	Natural pasture	AT26	1	nd		*A. chroococcum *	0.17	8.60	0.20	4.50
		AT27	1	I		*A. chroococcum *				
Entre Ríos (Paraná)	Wheat crop	AT39	1	nd		*A. chroococcum *	4.47	7.00	0.93	13.50
Córdoba (Corral de Bustos)	Wheat crop 1	AT32	2	nd		*A. armeniacus *	3.12	6.13	0.69	10.10
Córdoba (Corral de Bustos)	Wheat crop 2	AT33	2	II	HQ623182	*A. armeniacus *	3.48	6.08	0.63	11.60
Córdoba (Corral de Bustos)	Wheat crop 3	AT36	2	nd		*A. armeniacus *	3.15	6.06	0.52	11.10
Salta (Embarcación)	Soybean crop	AT19	3	IV	HQ591467	*A. salinestris *	1.78	6.40	0.21	48.80
Salta (Joaquín V. González)	Natural pasture	AT14	3	IV		*A. salinestris *	1.64	7.80	2.24	3.30
Santiago del Estero (Quimilí)	Soybean crop 1	AT1	3	nd		*A. salinestris *	3.10	7.17	nd	127.80
		AT2	3	nd		*A. salinestris *				
		AT16	3	nd		*A. salinestris *				
	Soybean crop 2	AT17	3	nd		*A. salinestris *	2.78	7.16	nd	104.20
		AT18	3	IV	HQ541448	*A. salinestris *				
Chubut (Puerto Madryn)	Natural pasture	AT42	4	IV	HQ623179	*A. salinestris *	1.09	7.50	0.48	7.40
Chubut (Villa Ameghino)	River bank	AT29	4	IV		*A. salinestris *	2.81	7.70	1.50	43.50
Jujuy (Tilcara)	Side of route^a^	AT12	5	IV		*A. salinestris *	0.19	8.77	0.28	4.80
Santa Fe (Videla)	Soybean crop 1	AT37	5	IV	HQ623178	*A. salinestris *	2.10	7.43	0.54	7.00
Santa Fe (Videla)	Soybean crop 2	AT38	5	IV		*A. salinestris *	1.00	8.28	0.94	3.00
Jujuy (Tilcara)	Side of route^a^	AT10	6	IV		*A. salinestris *	0.19	8.77	0.28	4.80

*Buenos Aires, Córdoba, Entre Ríos, and Santa Fe are provinces of the Pampas region; Jujuy, Salta, and Santiago del Estero are provinces from the Northwest region, Chubut is a province from Patagonia region of Argentina.

^a^Corresponded to the same soil sample.

**OM: organic matter; EC: electrical conductivity; P: extractable phosphorus; nd: not determined.

**Table 2:** Auxin production capacity of representative *Azotobacter* strains as determined by a colorimetric assay using the Salkowski reagent and results of rep-PCR clustering.

Strain	rep-PCR	Auxin production (*µ*g mL^−1^)			
	group	Day 1	Day 2	Day 3	Day 5
*A. chroococcum *AT9	1	1.80 ± 0.64^a^	2.50 ± 0.27^a^	4.13 ± 0.30^c^	4.23 ± 1.55^c^
*A. chroococcum *AT11	1	1.10 ± 0.41^a^	4.97 ± 1.27^a^	12.26 ± 0.25^a^	12.34 ± 2.07^b^
*A. chroococcum *AT13	1	1.41 ± 0.83^a^	4.58 ± 1.37^a^	11.38 ± 3.52^a^	12.72 ± 2.46^b^
*A. chroococcum *AT22	1	2.08 ± 0.09^a^	8.54 ± 2.02^a^	12.62 ± 0.61^a^	13.25 ± 1.54^b^
*A. chroococcum *AT31	1	1.38 ± 0.61^a^	9.30 ± 0.60^a^	12.68 ± 1.65^a^	14.76 ± 0.52^b^
*A. chroococcum *AT28	1	2.16 ± 0.81^a^	7.81 ± 0.44^a^	14.80 ± 0.10^a^	14.81 ± 0.93^b^
*A. chroococcum *AT25	1	2.64 ± 0.57^a^	7.97 ± 2.31^a^	12.82 ± 0.07^a^	15.33 ± 2.41^b^
*A. chroococcum *AT39	1	1.59 ± 0.40^a^	6.01 ± 1.09^a^	12.92 ± 1.40^a^	15.36 ± 0.03^b^
*A. chroococcum *AT30	1	1.40 ± 0.60^a^	4.97 ± 1.27^a^	12.86 ± 0.60^a^	16.11 ± 0.44^b^
*A. armeniacus* AT33	2	1.92 ± 0.32^a^	5.95 ± 0.29^a^	9.86 ± 0.45^b^	9.69 ± 1.06^b^
*A. salinestris* AT18	3	1.72 ± 0.27^a^	2.09 ± 1.08^a^	3.42 ± 0.21^c^	2.22 ± 1.59^c^
*A. salinestris* AT19	3	1.34 ± 0.09^a^	6.09 ± 0.43^a^	15.38 ± 0.68^a^	18.09 ± 0.98^a^
*A. salinestris* AT14	3	2.15 ± 0.08^a^	3.41 ± 0.69^a^	12.38 ± 0.37^a^	19.47 ± 0.48^a^
*A. salinestris* AT42	4	1.90 ± 0.33^a^	3.64 ± 0.30^a^	5.14 ± 0.76^c^	5.93 ± 1.69^c^
*A. salinestris* AT29	4	1.24 ± 0.76^a^	3.32 ± 0.60^a^	7.21 ± 0.40^b^	10.62 ± 1.73^b^
*A. salinestris* AT37	5	0.96 ± 0.55^a^	1.82 ± 0.08^a^	5.22 ± 1.28^c^	6.25 ± 0.08^c^
*A. salinestris* AT12	5	1.45 ± 0.60^a^	2.91 ± 0.43^a^	8.23 ± 0.12^b^	13.59 ± 1.30^b^
*A. salinestris* AT10	6	1.38 ± 0.13^a^	3.20 ± 0.72^a^	5.51 ± 0.15^c^	6.58 ± 2.38^c^

Auxin concentrations were determined after 1, 2, 3, and 5 days of growth in liquid culture. Values are means of two replicates ± standard error (*N* = 2). Same letters in a column indicate no significant differences as determined by the DGC test (*P* = 0.05).

**Table 3:** Effect of pure IAA solutions and *Azotobacter* inoculation on the number of seminal roots, and root colonization of 8-day-old wheat seedlings.

Treatment	IAA concentration *μ*g mL^−1^	Number of seminal roots per plant	Root colonization (cfu root^−1^)
Water		4.33 ± 0.12^c^	—
Low-IAA	2	4.69 ± 0.11^b^	—
High-IAA	20	5.03 ± 0.12^a^	—
*A. salinestris* AT18	2.2	4.68 ± 0.10^b^	4.38 × 10^4^ ± 1.68 × 10^4^ ^c^
*A. salinestris* AT37	2.5	4.74 ± 0.16^b^	6.94 × 10^5^ ± 2.67 × 10^5^ ^b^
*A. chroococcum *AT25	14.8	5.18 ± 0.18^a^	1.26 × 10^6^ ± 4.67 × 10^5^ ^a^
*A. chroococcum *AT31	14.2	5.33 ± 0.15^a^	8.41 × 10^5^ ± 1.87 × 10^5^ ^b^
*A. salinestris* AT19	18.2	5.39 ± 0.06^a^	3.01 × 10^6^ ± 1.12 × 10^6^ ^a^

Pregerminated seeds were treated with water (Control), IAA pure solutions of 2 *μ*g mL^−1^ (Low-IAA) and 20 *μ*g mL^−1^ (High-IAA), or were inoculated with *A. salinestris* strains (AT18, AT19, and AT37) or *A. chroococcum *strains (AT25 and AT31). Values are means ± standard error of two independent experiments with three replicates (*N* = 6). Different letters in a column indicate significant differences between means as determined by the DGC test (*P* = 0.05).

## References

[1] Kennedy C, Rudnick P, MacDonald M, Melton T, Garrity GM (2005). Genus III: Azotobacter. *Bergey's Manual of Systematic Bacteriology. The Proteobacteria, Part B, the Gammaproteobacteria*.

[2] Aquilanti L, Favilli F, Clementi F (2004). Comparison of different strategies for isolation and preliminary identification of *Azotobacter* from soil samples. *Soil Biology and Biochemistry*.

[3] Hussain A, Arshad M, Hussain A, Hussain E (1987). Response of maize (*Zea mays*) to *Azotobacter* inoculation under fertilized and unfertilized conditions. *Biology and Fertility of Soils*.

[4] Behl R, Narula N, Vasudeva M, Sato A, Shinano T, Osaki M (2006). Harnessing wheat genotype X *Azotobacter* strain interactions for sustainable wheat production in semi arid tropics. *Tropics*.

[5] Kizilkaya R (2008). Yield response and nitrogen concentrations of spring wheat (*Triticum aestivum*) inoculated with *Azotobacter chroococcum* strains. *Ecological Engineering*.

[6] Kennedy IR, Choudhury ATMA, Kecskés ML (2004). Non-symbiotic bacterial diazotrophs in crop-farming systems: can their potential for plant growth promotion be better exploited?. *Soil Biology and Biochemistry*.

[7] Kumar V, Narula N (1999). Solubilization of inorganic phosphates and growth emergence of wheat as affected by *Azotobacter chroococcum* mutants. *Biology and Fertility of Soils*.

[8] Khalid M, Zahir ZA, Waseem A, Arshad M (1999). *Azotobacter* and L-tryptophan application for improving wheat yield. *Pakistan Journal of Biological Science*.

[9] Pati BR, Sengupta S, Chandra AK (1995). Impact of selected phyllospheric diazotrophs on the growth of wheat seedlings and assay of the growth substances produced by the diazotrophs. *Microbiological Research*.

[10] Zahir ZA, Asghar HN, Akhtar MJ, Arshad M (2005). Precursor (L-tryptophan)-inoculum (*Azotobacter*) interaction for improving yields and nitrogen uptake of maize. *Journal of Plant Nutrition*.

[11] Boraste A, Vamsi KK, Jhadav A (2009). Biofertilizers: a novel tool for agriculture. *International Journal of Microbiology Research*.

[12] Sparks DL, Page AL, Helmke PA (1996). Chemical methods. *Methods of Soil Analysis*.

[13] Versalovic J, Schneider M, de Bruijn FJ, Lupski JR (1994). Genomic fingerprinting of bacteria using repetitive sequence-based polymerase chain reaction. *Methods in Molecular and Cellular Biology*.

[14] Montecchia MS, Kerber NL, Pucheu NL, Perticari A, García AF (2002). Analysis of genomic diversity among photosynthetic stem-nodulating rhizobial strains from Northeast Argentina. *Systematic and Applied Microbiology*.

[15] Young JPW, Downer HL, Eardly BD (1991). Phylogeny of the phototrophic *Rhizobium* strain BTAi1 by polymerase chain reaction-based sequencing of a 16S rRNA gene segment. *Journal of Bacteriology*.

[16] Altschul SF, Madden TL, Schäffer AA (1997). Gapped BLAST and PSI-BLAST: a new generation of protein database search programs. *Nucleic Acids Research*.

[17] Pérez-Miranda S, Cabirol N, George-Téllez R, Zamudio-Rivera LS, Fernández FJ (2007). O-CAS, a fast and universal method for siderophore detection. *Journal of Microbiological Methods*.

[18] Pikovskaya RI (1948). Mobilization of phosphorus in soil in connection with vital activity of some microbial species. *Microbiologiya*.

[19] Nautiyal CS (1999). An efficient microbiological growth medium for screening phosphate solubilizing microorganisms. *FEMS Microbiology Letters*.

[20] Glickmann E, Dessaux Y (1995). A critical examination of the specificity of the Salkowski reagent for indolic compounds produced by phytopathogenic bacteria. *Applied and Environmental Microbiology*.

[21] Perrig D, Boiero ML, Masciarelli OA (2007). Plant-growth-promoting compounds produced by two agronomically important strains of *Azospirillum brasilense*, and implications for inoculant formulation. *Applied Microbiology and Biotechnology*.

[22] Di Rienzo JA, Guzmán AW, Casanoves F (2002). A multiple-comparisons method based on the distribution of the root node distance of a binary tree. *Journal of Agricultural, Biological, and Environmental Statistics*.

[23] Di Rienzo JA, Casanoves F, Balzarini MG, Gonzalez L, Tablada M, Robledo CW *InfoStat Versión 2010*.

[24] Aquilanti L, Mannazzu I, Papa R, Cavalca L, Clementi F (2004). Amplified ribosomal DNA restriction analysis for the characterization of Azotobacteraceae: a contribution to the study of these free-living nitrogen-fixing bacteria. *Journal of Microbiological Methods*.

[25] Page WJ, Shivprasad S (1991). *Azotobacter salinestris* sp. nov., a sodium-dependent, microaerophilic, and aeroadaptive nitrogen-fixing bacterium. *International Journal of Systematic Bacteriology*.

[26] Thompson JP, Skerman VBD (1979). *Azotobacteraceae: The Taxonomy and Ecology of the Aerobic Nitrogen-Fixing Bacteria*.

[27] Farajzadeh D, Yakhchali B, Aliasgharzad N, Sokhandan-Bashir N, Farajzadeh M (2012). Plant growth promoting characterization of indigenous *Azotobacteria* isolated from soils in Iran. *Current Microbiology*.

[28] Cavaglieri L, Orlando J, Etcheverry M (2005). *In vitro* influence of bacterial mixtures on *Fusarium verticillioides* growth and fumonisin B1 production: effect of seeds treatment on maize root colonization. *Letters in Applied Microbiology*.

[29] Ahmad F, Ahmad I, Khan MS (2008). Screening of free-living rhizospheric bacteria for their multiple plant growth promoting activities. *Microbiological Research*.

[30] Kukreja K, Suneja S, Goyal S, Narula N (2004). Phytohormone production by Azotobacter—a review. *Agricultural Reviews*.

[31] Spaepen S, Dobbelaere S, Croonenborghs A, Vanderleyden J (2008). Effects of *Azospirillum brasilense* indole-3-acetic acid production on inoculated wheat plants. *Plant and Soil*.

[32] Taiz L, Zeiger E, Taiz L, Zeiger E (1998). The biological roles of cytokinins. *Cytokinins. Plant Physiology*.

