# Supplementary material for: Genotypic Characterization of Azotobacteria Isolated from Argentinean Soils and Plant-Growth-Promoting Traits of Selected Strains with Prospects for Biofertilizer Production
Source: ScientificWorldJournal. 2013 Nov 3;2013:519603. doi: 10.1155/2013/519603 (PMC3835881; doi:10.1155/2013/519603)
Supplement: Supplementary file 1 — Supplementary Table: Complete list of all soil samples analyzed. Geographical origin and coordinates, land use, number of Azotobacter-like isolates obtained per soil sample, and soil characteristics. [file 519603.f1.pdf]

Supplementary material

Complete list of all soil samples analyzed. Geographical origin and coordinates, land use, number of *Azotobacter*-like isolates obtained per soil sample, and soil characteristics

Sample	Geographical origin*	Geographical coordinates	Sampling site	Number of isolates	Soil chemical parameters**			
					MO (%)	pH	EC (mS cm^{-1})	P (ppm)
			Agricultural soils					
1	Buenos Aires (Azul)	S 36° 46' 27.5" W 59° 51' 15.2"	Maize crop	-	5.12	5.50	0.15	14.90
2	Buenos Aires (Azul)		Maize stubble	1	3.38	7.30	0.48	7.40
3	Buenos Aires (Pergamino)	S 33° 52' 59.7" W 60° 34' 11.1"	Soybean crop	-	4.79	5.30	0.88	72.10
4	Buenos Aires (Pergamino)		Wheat crop	-	4.02	6.00	1.35	48.60
5	Buenos Aires(Tres Arroyos)	S 38° 22' 34.0" W 60° 16' 43.5"	Sunflower crop	-	3.47	5.41	0.79	34.80
6	Buenos Aires(Necochea)	S 38° 33' 11.8" W 58° 44' 23.4"	Oat crop	-	4.36	6.27	0.20	30.30
7	Buenos Aires (San Antonio de Areco)	S 34° 14' 53.3" W 59° 27' 58.6"	Wheat crop	-	3.28	5.45	0.80	18.60
8	Buenos Aires (Orense)	S 38° 41' 9.0" W 59° 46' 21.2"	Wheat crop	-	4.90	5.40	0.75	17.10
9	Buenos Aires (Balcarce)	S 37° 50' 44.6" W 58° 15' 21.2"	Agricultural bare	1	5.72	5.80	1.21	51.00
10	Buenos Aires(Tres Arroyos)		Wheat crop	-	3.34	5.20	0.22	10.40
11	Buenos Aires (Chivilcoy)	S 34° 53' 46.3" W 60° 1' 8.5"	Soybean crop	-	3.81	5.50	0.33	6.70
12	Buenos Aires (Chivilcoy)		Soybean crop	-	2.38	5.50	0.26	3.60
13	Buenos Aires (Chivilcoy)		Soybean crop	-	1.10	5.90	0.43	2.10
14	Buenos Aires (Chivilcoy)		Soybean crop	-	4.10	5.40	0.39	7.30
15	Buenos Aires (Chivilcoy)		Soybean crop	-	3.53	5.50	0.26	5.90
16	Buenos Aires (Chivilcoy)		Soybean crop	-	1.26	5.90	0.30	1.30
17	Buenos Aires (Ramallo)	S 33º 31' 24" W 59º 59' 53"	Sunflower crop	-	3.31	nd	nd	18.90
18	Buenos Aires (Esteban Echeverría)	S 34° 52' 23.2" W 58° 28' 29.7"	Agricultural bare	-	2.57	5.69	0.53	8.60
19	Buenos Aires (Castelar)	S 34° 38' 32.7" W 58° 42' 23.3"	Wheat stubble	-	3.02	5.62	0.52	15.80
20	Buenos Aires (Junín)	S 34° 35' 12.7" W 60° 56' 58.0"	Soybean crop	-	2.07	6.1	0.42	3.40
21	Buenos Aires (Junín)		Soybean crop	-	0.86	7.35	0.32	2.10
22	Buenos Aires (Junín)		Soybean crop	-	0.12	8.92	0.33	3.10
23	Córdoba (Corral de Bustos)	S 33° 16' 59.1" W 62° 11' 7.8"	Wheat crop	-	3.10	5.81	0.68	24.10
24	Córdoba (Corral de Bustos)		Wheat crop	-	2.90	6.00	0.55	11.30
25	Córdoba (Corral de Bustos)		Wheat crop	-	3.00	5.93	0.63	21.10

26	Córdoba (Corral de Bustos)		Wheat crop	-	2.86	6.20	0.57	7.40
27	Córdoba (Corral de Bustos)		Wheat crop	1	3.12	6.13	0.69	10.10
28	Córdoba (Corral de Bustos)		Wheat crop	1	3.48	6.08	0.63	11.60
29	Córdoba (Corral de Bustos)		Wheat crop	1	3.15	6.06	0.52	11.10
30	Córdoba (Huinca Renancó)	S 34° 50' 24.8" W 64° 22' 19.0"	Soybean crop	-	1.34	6.33	0.4	31.60
31	Córdoba (Santa Eufemia)	S 33° 10' 38" O 63° 17' 20.1"	Peanut crop	-	2.16	5.1	1.21	41.70
32	Córdoba (Santa Eufemia)		Wheat crop	-	3.57	5.53	0.72	31.30
33	Córdoba (Bell Ville)	S 32° 37' 11.2" W 62° 41' 21.3"	Soybean crop	-	2.02	6.13	0.65	16.50
34	Córdoba (Bell Ville)		Soybean crop	-	2.02	6.13	0.65	16.50
35	Entre Ríos (Paraná)	S 31° 44' 17.1" W 60° 49' 24.1"	Wheat crop	1	4.47	7.00	0.93	13.50
36	Santa Fe (Videla)	S 30° 56' 0.4" O 60° 39' 2.9"	Soybean crop	1	2.10	7.43	0.54	7.00
37	Santa Fe (Videla)		Soybean crop	-	1.59	8.07	1.04	3.10
38	Santa Fe (Videla)		Soybean crop	1	1.00	8.28	0.94	3.00
39	Santa Fe (Videla)		Soybean crop	-	2.45	6.53	0.75	18.00
40	Santa Fe (Videla)		Soybean crop	-	1.79	6.38	0.28	6.40
41	Santa Fe (Videla)		Soybean crop		1.12	6.71	0.46	5.30
42	Santa Fe (Runciman)	S 33° 53' 39.7" W 61° 49' 24.1"	Soybean crop	-	3.43	5.00	1.26	33.50
43	Santa Fe (Runciman)		Soybean crop	-	4.64	5.00	1.45	67.00
44	Santa Fe (Amstrong)	S 32° 46' 52.5" W 61° 36' 8.8"	Soybean crop	-	3.36	6.00	0.71	101.40
45	Santa Fe (Amstrong)		Soybean crop	-	3.41	6.00	0.67	76.10
46	Santa Fe (Amstrong)		Soybean crop	-	3.45	6.10	0.39	40.20
47	Santa Fe (Oliveros)	S 32° 34' 27.4" W 60° 50' 59.8"	Soybean crop	-	1.88	5.63	0.51	12.10
48	Santa Fe (Venado Tuerto)	S 33 44 58.1 W 61 57 55.3	Soybean crop	-	3.74	6.07	0.44	35.80
49	Santiago del Estero (Quimilí)	S 27° 38' 37.6" W 62° 24' 56.2"	Soybean crop	-	2.84	7.03	nd	125.70
50	Santiago del Estero (Quimilí)		Soybean crop	3	3.10	7.17	nd	127.80
51	Santiago del Estero (Quimilí)		Soybean crop	2	2.78	7.16	nd	104.20
52	Salta (Embarcación)	S 23° 12' 32.1" W 64° 5' 50.1"	Soybean crop	1	1.78	6.40	0.21	48.80
			Non-agricultural soils					
53	Buenos Aires (Arrecifes)	S 34° 2' 48.1" W 60° 6' 13.5"	Clover pasture	-	4.36	4.70	0.53	29.70
54	Buenos Aires (Santa Clara del Mar)	S 37° 46' 45.8" W 57° 30' 54.5"	Side of road	1	5.72	7.83	0.80	8.50
55	Buenos Aires (Santa Clara del Mar)		Urban land	2	0.98	8.45	0.48	8.50
56	Buenos Aires (Vedia)	S 34° 39' 53.4" W 61° 32' 56.2"	Clover pasture	-	2.95	5.50	0.29	15.00
57	Buenos Aires (Mar Chiquita)	S 37° 44' 31.0" W 57° 25' 49.6"	Lagoon bank 1	1	1.86	8.20	0.43	1.90

58	Buenos Aires (Mar Chiquita)		Lagoon bank 2	1	1.05	8.00	1.45	7.70
59	Buenos Aires (Bordenave)	S 37° 48' 12.8" W 63° 2' 28.3"	Fescue pasture	-	3.38	6.99	0.17	11.10
60	Buenos Aires (Bordenave)		Agropyron pasture	-	6.79	6.40	0.24	11.40
61	Buenos Aires (Necochea)		Side of road	-	7.07	6.05	0.22	146.40
62	Santa Fe (Runciman)		Natural pasture	-	4.55	nd	nd	72.20
63	Santa Fe (Runciman)		Natural pasture	-	4.24	nd	nd	79.40
64	Chubut (Puerto Madryn)	S 42° 45' 4.7" W 65° 2' 55.4"	Natural pasture	1	1.09	7.50	0.48	7.40
65	Chubut (Gaiman)	S 43° 17' 20.0" W 65° 29' 31.8"	Natural pasture	1	3.15	8.30	0.66	45.80
66	Chubut (Villa Ameghino)	S 43° 47' 57.7" W 65° 44' 57"	River bank	1	2.81	7.70	1.50	43.50
67	Chubut (Trevelín)	S 43° 4' 44.7" W 71° 27' 51.7"	River bank	1	1.02	6.60	1.58	8.10
68	Chubut (Esquel)	S 42° 55' 1.5" W 71° 19' 20.7"	Natural pasture	1	2.74	6.40	0.49	40.40
69	Neuquén (Villa La Angostura)	S 40° 45' 47.8" W 71° 38' 46.1"	Creek edge	-	1.79	6.20	1.51	17.50
70	Río Negro (Gutiérrez)	S 38° 56' 9.9" W 67° 58' 19.2"	Creek edge	-	8.03	6.20	nd	45.50
71	Río Negro (Gutiérrez)		Natural pasture	-	2.74	5.40	1.48	1.00
72	Salta (J.V. González)	S 25° 6' 53.9" W 64° 7' 48.2"	Natural pasture	1	1.64	7.80	2.24	3.00
73	Jujuy (Tilcara)	S 24° 11' 58.5" W 65° 17' 55.1"	Side of road	4	0.19	8.77	0.28	4.80
74	Jujuy (Tilcara)		Natural pasture	2	0.17	8.60	0.20	4.50

Buenos Aires, Córdoba, Entre Ríos and Santa Fe are provinces from Pampas region, Jujuy, Salta and Santiago del Estero are provinces from Northwest region, and Chubut, Neuquén and Río Negro are provinces from Patagonia region of Argentina.

** OM: organic matter; EC: electrical conductivity; P: extractable phosphorus; nd: not determined
